# Frequency, duration and predictors of bronchiolitis episodes of care among infants ≥32 weeks gestation in a large integrated healthcare system: a retrospective cohort study

**DOI:** 10.1186/1472-6963-12-144

**Published:** 2012-06-08

**Authors:** Valerie J Flaherman, Arona I Ragins, Sherian Xu Li, Patricia Kipnis, Anthony Masaquel, Gabriel J Escobar

**Affiliations:** 1 Department of Pediatrics, University of California, 3333 California St, Box 0503, San Francisco, CA, 94143-0503, USA; 2 Division of Research, Kaiser Permanente Medical Care Program, Systems Research Initiative and Perinatal Research Unit, 2000 Broadway, 2nd floor, Oakland, CA, 94612, USA; 3 Kaiser Foundation Health Plan, Inc, Management Information and Analysis, 1950 Franklin St, 17th floor, Oakland, CA, 94612, USA; 4 MedImmune, LLC, One MedImmune Way, Gaithersburg, MD, 20878, USA; 5 Department of Inpatient Pediatrics, Kaiser Permanente Medical Center, 1425 S. Main St, Walnut Creek, CA, 94596, USA

## Abstract

**Background:**

Bronchiolitis is common in the first two years of life and is the most frequent cause of hospitalization in this age group. No previous studies have used an episode-of-care analysis to describe the frequency, duration, and predictors of bronchiolitis episodes of care during the first two years.

**Methods:**

We conducted a retrospective cohort study of 123,264 infants ≥32 weeks gestation born at 6 Northern California Kaiser Permanente hospitals between 1996 and 2002. We used electronic medical records to concatenate hospital, emergency department and outpatient health care encounters for bronchiolitis into discrete episodes of care. We used descriptive statistics to report frequency and duration of bronchiolitis episodes and used logistic regression to assess the effect of gestational age and other clinical and demographic predictors on the outcome of bronchiolitis episodes.

**Results:**

Among all infants, the rate of bronchiolitis episodes was 162 per 1000 children during the first 2 years of life; approximately 40% required >1 day of medical attention with a mean duration of 7.0 ± 5.9 days. Prematurity was associated with increased risk of bronchiolitis episodes and longer duration. Bronchiolitis episodes rates per 1000 infants were 246 for 32–33 weeks gestational age, 204 for 34–36 weeks, and 148–178 for >36 weeks. Male gender, African-American and Hispanic race/ethnicity, and parental history of asthma were associated with an increased risk of having a bronchiolitis episode and/or longer duration.

**Conclusions:**

Bronchiolitis episodes of care are frequent during the first two years of life and the duration ranges from 1 to 27 days. Prematurity was associated with more frequent and longer duration of bronchiolitis episodes of care, which may reflect illness severity and/or perceived vulnerability.

## Background

Bronchiolitis is the most common lower respiratory condition in children younger than 2 years [1], and presents with a wide spectrum of clinical illness, from mild symptoms to severe, life-threatening respiratory distress. More severe bronchiolitis is the most frequent cause of infant hospitalization in the United States [2,3] with costs estimated at $543 million per year [4]. Bronchiolitis also causes a substantial emergency department (ED) and outpatient burden [5-7]. Populations at increased risk for severe bronchiolitis include racial and ethnic minorities and those who had preterm birth, bronchopulmonary dysplasia (BPD) or congenital heart disease. [6,8,9]

Previous research on the epidemiology of bronchiolitis has focused on ascertaining rates of outpatient care, ED visits, and hospitalizations, with widely differing results that can be attributed to differences in study designs, methodologies, and populations. Estimates of the rate of outpatient visits for children <2 years range from 103 per 1000 children [2] to 238 visits per 1000 child years [5]. Estimates of the rate of hospitalization also vary. Koehoorn et al. [10] found a hospitalization rate of 17.1 per 1000 child years for children <1 year. Shay et al., estimated similar rates of hospitalization in the US for infants <1 year ranging from 12.9 per 1000 in 1980 to 31.2 per 1000 in 1996 [11], whereas Carroll et al. [5] estimated a higher rate of 71 per 1000 child years during 1995–2003. Carroll et al. [5] also estimated a rate of 77 ED visits per 1000 child years. Respiratory syncytial virus is an important cause of bronchiolits, and recent research has examined the prevalence of respiratory syncytial virus (RSV) among children presenting for medical care, and found that 15-20% of children presenting for care had RSV infection or co-infection [12].

These existing studies have tabulated the frequency of hospitalizations and individual outpatient visits for bronchiolitis, and have estimated the frequency of RSV infection among those receiving care, but have not provided a comprehensive description of the healthcare utilization for each infant over a specific time period. In order to provide such a comprehensive description, health care effectiveness researchers have promoted “episode of care” analysis. Hornbrook et al. [13] first defined an episode as a “series of temporally contiguous health services related to treatment of a given spell of illness or provided in response to a specific request by the patient.” Recently episode analysis has been used as an important tool for estimating costs, determining patterns of use, evaluating outcomes of care, and understanding medical practice variation [13-15], using the entire duration of care as the basis for measurement, with the assumption that this will be more effective than counting single outpatient visits or single hospitalizations [13,14,16]. To the best of our knowledge, no previous studies have defined episodes of care for bronchiolitis or have reported their frequency, duration or risk during the first 2 years of life.

In this study, we use an “episodes of care” (henceforth, “episode” or EOC) approach to determine the frequency, duration and risk factors for bronchiolitis in children <2 years among members of a large, integrated healthcare system in Northern California. Using this approach, we analyze our results by gestational age (GA) to examine the burden of disease among those born preterm, who are considered at high risk for bronchiolitis [17]. Below we describe the frequency, duration and characteristics of bronchiolitis episodes and the clinical and demographic predictors of bronchiolitis episodes longer than one day among preterm and term infants.

## Methods

### Study design

**Figure 1:**
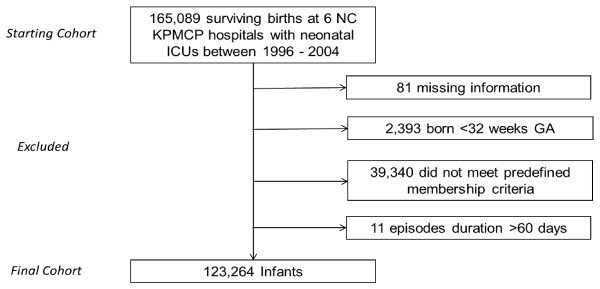
Cohort Selection Process.

This study was approved by the Kaiser Permanente Medical Care Program (KPMCP) Institutional Review Board for the Protection of Human Subjects, which has jurisdiction over all the hospitals and clinics described in this study. We conducted a retrospective cohort study that included infants ≥32 weeks GA born and discharged alive from one of 6 Northern California KPMCP hospitals between 1996 and 2004. Infants <32 weeks GA at birth represent a large portion of all infants who receive RSV prophylaxis in our integrated healthcare system and represent only a small portion of all preterm infants born annually [18]. Consequently, infants <32 weeks GA were not included in this study due to routine administration of RSV immunoprophylaxis. To reduce the effects of loss to follow up on our analysis we identified those infants who remained KPMCP members until they were two years of age. We included subjects in the cohort if they were members for ≥20 of the 24 months after the infant’s birth, and if they had no more than two consecutive months without membership. Subjects were defined as members for each month in the study if they were either insured by KPMCP or used KPMCP medical services. See Figure 1 for flowchart of the cohort selection.

We extracted data regarding these infants from KPMCP databases, including inpatient and outpatient encounters and diagnoses. Because there may be some use of KPMCP facilities without membership, we conducted a sensitivity analysis examining our results among a subcohort of infants who were recorded as members for all 24 months after the infant’s birth (see Additional file 1: Tables S1, S 2, and S 3). The race/ethnicity of participants was based on the self-reported race/ethnicity of infants' mothers.

### Variables and measurements

To identify only episodes that included a true diagnosis of bronchiolitis while capturing all relevant clinical information associated with a bronchiolitis episode, we classified relevant International Classification of Diseases (ICD-9) codes into two categories. Category A included diagnostic codes specific for bronchiolitis in children younger than two years (ie, 466.1 acute bronchiolitis, 466.1x acute bronchiolitis organism specified, 466.0 acute bronchitis, 480.0–480.2 pneumonia due to adenovirus, RSV or parainfluenza, 079.0 adenovirus and 079.6 RSV). Category B included diagnostic codes likely to be highly related to bronchiolitis in a child with a current diagnosis of bronchiolitis (i.e., 786.06 tachypnea, 786.07 wheezing, 786.09 other respiratory distress, 780.6–780.60, 786.2 cough, 460 acute nasopharyngitis, 465 acute upper respiratory infections, 465.9 acute upper respiratory infection not otherwise specified, and 487.1 acute upper respiratory infection due to influenza.)

We defined a bronchiolitis episode as a period that included a diagnostic code specific for bronchiolitis from Category A, began either with the first bronchiolitis-related diagnostic code from Category B occurring within 2 days prior to the first Category A code or with the first Category A code if there was no Category B code within 2 days prior to the first Category A code, and ended with a diagnostic code from either Category A or Category B followed by 14 clear days without a bronchiolitis or related diagnosis. An episode of care can include multiple healthcare visits and/or various types of healthcare visits, including hospitalization or outpatient visits with the aforementioned category codes. The duration of an episode of care is defined as the length of time between the first and last visit.

For all infants, we extracted data regarding additional clinical predictors including GA, sex, race/ethnicity, maternal age, birth weight, a diagnosis of BPD or congenital anomaly (ICD-9 codes 425.3x, 425.4x, 425.8x, 745.xx, 746.xx, 747.xx) and the number of siblings present in the home (≥1 sibling <5 years of age). For hospitalization records, we also determined whether the infant required assisted ventilation (ICD-9 codes 93.90, 93.91, and 96.7x).

We classified infants as being small for gestational age (SGA) (<5th percentile) using birth weight and GA according to the algorithm developed by Brenner et al. [19]. We fitted a smooth cubic spline as a function of total oxygen exposure separately for births occurring at 32–37 and ≥37 weeks GA. Both curves showed a flat effect for total oxygen exposure <200 hours and a clear increase at >200 hours, suggesting a step function and no interaction between GA and total length of oxygen therapy. For infants treated in the neonatal intensive care unit, we created an oxygen exposure variable: no supplemental oxygen exposure during the neonatal period, no bronchopulmonary dysplasia (BPD); supplemental oxygen exposure of 1 to <200 hours during the neonatal period, no BPD; supplemental exposure ≥200 hours during the neonatal period, no BPD; and BPD. We classified infants who did not require intensive care during the neonatal period as having no supplemental oxygen exposure.

Parental asthma history was established by scanning the parent’s records for the period 18 months before to 6 months after the infant’s birth and determining whether the parent had ≥2 clinical visits 14 days apart with an ICD-9 code (493.xx) for asthma, and/or the parent’s electronic record listed asthma on their Significant Problem List.

### Statistical analysis

All statistical analyses used SAS (version 9.1, SAS Institute, Inc., Cary, NC) or Stata (version 9.2, Stata Corp., College Station, TX). After identifying duration of episode, we eliminated episodes lasting >60 days from further analysis because it was unlikely that these represented isolated bronchiolitis (see Additional file 1: Table S4). Manual chart review of infants with episodes lasting >60 days confirmed that these episodes represented hospitalizations for which bronchiolitis was not the primary diagnosis. Infants remaining after this step constituted the final analysis cohort. We used descriptive statistics to report frequency and duration of bronchiolitis episodes and frequency of hospitalization associated with the episode, by age at the episode, gender, GA, and race/ethnicity. We used a moving average of GA and GA minus 1 week to develop a graph of mean, 10th percentile and 90th percentile for the duration of EOC for each GA. We used multivariable logistic regression to assess the effect of GA, gender, race/ethnicity, family history of asthma, neonatal oxygen use and other clinical predictors on the outcome of whether an infant had a documented bronchiolitis EOC and on the outcome of whether an infant had a documented bronchiolitis EOC lasting >1 day. We used White race/ethnicity as the referent category for this multivariate analysis because this was the largest population in our cohort and because previous literature has described disparate risk of bronchiolitis for non-White racial/ethnic groups. We calculated the relative contribution of each predictor using the differences between the log likelihood of the full model and the log likelihood of a model without each of the predictors, and the relative contribution of each predictor was defined as the ratio of its log likelihood difference to the sum of the likelihood differences from all predictors × 100 [20].

## Results

We selected our cohort from 165,089 surviving births during 1996 to 2004 at the six Northern California KPMCP hospitals equipped with neonatal intensive care units. From this original birth population we removed 81 infants with missing information, 2,393 infants born <32 weeks GA, 39,340 infants who did not meet predefined membership criteria, and an additional 11 infants with episode duration >60 days, resulting in a final cohort of 123,264 infants. The demographics and clinical characteristics of the cohort are presented in Table 1.

**Table 1:** Demographic and clinical characteristics of the main cohort and subgroups

	**All infants, n (%)**	**Infants with any bronchiolitis episode before age 2 y, n (%)**	**Infants with any bronchiolitis episode before age 2 y lasting >1 d, n (%)**
Sex (Male)	63,054 (51.2)	11,730 (58.7)	5,206 (60.1)
Race^a^			
White	52,609 (42.7)	8,305 (41.6)	3,754 (43.4)
African-American	10,374 (8.4)	1,866 (9.3)	740 (8.6)
Asian	24,977 (20.3)	3,599 (18.0)	1,505 (17.4)
Hispanic	25,619 (20.8)	4,704 (23.6)	2,047 (23.6)
Other/unknown	9,685 (7.9)	1,494 (7.5)	612 (7.1)
Gestational age, wk			
32–33	1,649 (1.3)	406 (2.0)	218 (2.5)
34–36	7,969 (6.5)	1,622 (8.1)	830 (9.6)
37	7,855 (6.4)	1,400 (7.0)	632 (7.3)
38–40	86,708 (70.3)	13,701 (68.6)	5,834 (67.4)
≥41	19,083 (15.5)	2,839 (14.2)	1,144 (13.2)
Small for gestational age^b^	1,763 (1.4)	323 (1.6)	168 (1.9)
Congenital anomaly present^c^	8,646 (7.0)	1,642 (8.2)	764 (8.8)
Family history of asthma^d^			
None	116,763 (94.7)	18,618 (93.2)	8,011 (92.5)
Father only	1,824 (1.5)	336 (1.7)	160 (1.9)
Mother only	4,561 (3.7)	989 (5.0)	476 (5.5)
Both parents	116 (0.1)	25 (0.1)	11 (0.1)
≥1 sibling <5 y of age in home	44,235 (35.9)	8,405 (42.1)	3,801 (43.9)
O_2_ exposure and BPD^e^			
No O_2_ exposure, no BPD	118,599 (96.2)	18,941 (94.9)	8,126 (93.9)
<200 hr O_2_, no BPD	4,117 (3.3)	867 (4.3)	427 (4.9)
≥200 hr O_2_, no BPD	466 (0.4)	138 (0.7)	89 (1.0)
BPD^f^	82 (0.1)	22 (0.1)	16 (0.2)

BPD = bronchopulmonary dysplasia.

^a^Classification of race/ethnicity in this study was based on the race of infants' mothers, as self-reported to the birth certificate clerk interviewing them at the Kaiser Permanente hospitals described in this study. Race was assessed in this study because of the well-known differences in RSV severity by race.

^b^Based on the algorithm of Brenner WE, et al. [19].

^c^See text for list of included International Classification of Diseases codes.

^d^Ascertained by electronic scanning of parental records, which included encounters and diagnoses identified in the Significant Problem List by the parent’s physician.

^e^Oxygen exposure, in hours, during the birth hospitalization. See text for details.

^f^All infants with BPD had ≥200 hours of oxygen exposure during the neonatal period.

Our main analysis focused on bronchiolitis episodes, which included ≥1 medically attended visit for bronchiolitis. Among all infants (123,264), 19,968 infants developed ≥1 episode for bronchiolitis during their first two years of life. Overall, there were 23,748 episodes identified throughout the study period. Among infants who experienced an episode, the mean number of episodes per infant was 1.2 within the first two years of life, so that 16.2% of infants eventually developed bronchiolits. The overall mean (± SD) duration of episodes was 3.2 ± 4.7 days. Overall, 2,036 (8.6%) episodes included a hospitalization. Of note, 6,351 (26.7%) included a diagnosis of otitis media.

Among episodes lasting >1 day (40%), the mean duration was 7.0 ± 5.9 days; of all episodes, 13.4% lasted 2–3 days, 11.3% lasted 4–6 days, 10.0% lasted 7–13 days and 5.0% lasted ≥14 days. The mean number of outpatient visits among episodes lasting >1 day was 2.3, while the mean number of emergency department (ED) visits was 0.2. The majority (85.2%) of episodes with an ED visit for bronchiolitis had no other healthcare encounters during the episode. However, among children who had an ED visit for bronchiolitis, the mean episode length was 6.3 days and the proportion of infants admitted to the hospital was 35.6% (n = 626).

**Figure 2:**
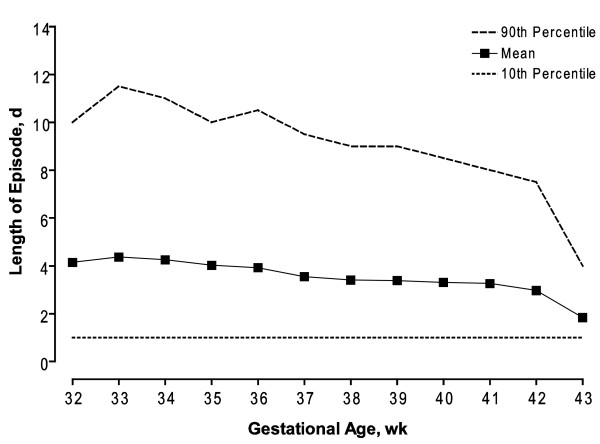
Bronchiolitis episode of care duration as a function of gestational age.

Preterm birth was associated with both frequency and duration of bronchiolitis episodes (Table 2). The episode rate and corresponding mean duration increased with lower GA. The rate during the first two years of life and mean duration of bronchiolitis EOC were 246 per 1000 infants and 4.4 ± 5.5 days, respectively, for infants born at 32–33 weeks and 204 per 1000 infants and 3.9 ± 5.0 days, respectively, for infants born at 34–36 weeks GA. In contrast, the rate of episodes among full term infants was lower, 149–178 per 1000 infants. Infants of 32–33 weeks gestational age were also twice as likely as term infants to have a bronchiolitis episode that included an ED visit and a hospitalization (14.2% vs. 6.4%). Figure 2 shows the increased trend in duration of bronchiolitis episodes in infants with lower GA. We also examined episodes by chronologic age. Overall, 10,380 (43.7%) episodes occurred in infants <6 months of age. For infants <6 months of age, the proportion of bronchiolitis episodes including a hospitalization was 11.5%; this proportion was 6% for those aged 6–24 months.

**Table 2:** Episode frequency and duration and associated hospitalization during the first two years of life

**Gestational age, wk**	**Infants, n**	**Infants with at least one EOC, n**	**Rate of at least one episode per thousand children**	**EOC**^**a**^**, n**	**Mean episode duration, d (SD)**	**Episode consisting of ED visit and hospitalization and lasting >1 d**^**b**^**, n (%)**	**Episode with any hospitalization, n (%)**
32–33	1,649	406	246.2	523	4.4 (5.5)	37 (14.2)	101 (19.3)
34–36	7,969	1,622	203.5	2,003	3.9 (5.0)	72 (7.8)	254 (12.7)
37	7,855	1,400	178.2	1,681	3.4 (4.6)	44 (6.4)	151 (9.0)
38–40	86,708	13,701	158.0	16,172	3.3 (4.7)	396 (6.3)	1,288 (8.0)
≥41	19,083	2,839	148.8	3,369	3.2 (4.7)	70 (5.6)	242 (7.2)
Total	123,264	19,968	162.0	23,748	3.2 (4.7)	619 (6.6)	2,036 (8.6)

ED = emergency department. EOC = Episode of Care.

^a^Across episodes during the first two years of life, there were a total of 35,703 outpatient visits for a rate of 289 per 1000 children, a total of 1909 emergency department visits for a rate of 15 per 1000 children and 2169 hospitalizations for a rate of 17.5 per 1000 children.

^b^Denominator is among episodes >1 day for each gestational age cohort.

In our multivariable model, demographic characteristics of male gender and African-American and Hispanic race/ethnicity were associated with increased risk of any episode with odds ratios (OR) of 1.44 (95% CI, 1.39–1.48), 1.19 (95% CI, 1.12–1.25) and 1.20 (95% CI, 1.15–1.25), respectively, while adjusting for confounding variables (Table 3). In the same model, Asian race/ethnicity and maternal age ≥35 years were associated with decreased risk of a bronchiolitis episode with OR of 0.91 (95% CI, 0.87–0.95) and 0.90 (95% CI, 0.86–0.93), respectively. Gestational age was an important predictor of any bronchiolitis episode, with infants at 32–33 weeks GA having an OR of 1.57 (95% CI, 1.39–1.77), infants 34–36 weeks GA having an OR of 1.32 (95% CI, 1.24–1.40), infants at 37 weeks GA having an OR of 1.15 (95% CI, 1.08–1.22), and infants ≥41 weeks GA having an OR of 0.95 (95% CI, 0.90–0.99) for the outcome of any episode when compared with infants 38–40 weeks GA. Other significant risk factors for bronchiolitis episodes included small for GA, presence of congenital anomaly, family history of asthma, at least one sibling <5 years of age in the home, and degree of oxygen exposure or having BPD.

**Table 3:** Effect of demographic and clinical predictors on eventual bronchiolitis episodes in multivariate analysis

**Clinical and demographic predictors**	**Beta**	**Odds Ratio (95% CI)**
Sex (Male)	0.36	1.44 (1.39–1.48)
Race^a^		
White	1.00	reference
African-American	0.17	1.19 (1.12–1.25)
Asian	−0.10	0.91 (0.87–0.95)
Hispanic	0.18	1.20 (1.15–1.25)
Other/unknown	−0.01	0.99 (0.93–1.05)
Gestational age, wk		
32–33	0.45	1.57 (1.39–1.77)
34–36	0.27	1.32 (1.24–1.40)
37	0.14	1.15 (1.08–1.22)
38–40	1.00	reference
≥41	−0.06	0.95 (0.90–0.99)
Small for gestational age^b^	0.16	1.17 (1.03–1.32)
Congenital anomaly present^c^	0.13	1.14 (1.08–1.21)
Family history of asthma^d^		
None	1.00	
Father only	0.16	1.17 (1.04–1.32)
Mother only	0.37	1.45 (1.35–1.56)
Both parents	0.38	1.46 (0.94–2.29)
≥1 sibling <5 y of age in home	0.33	1.39 (1.35–1.44)
O_2_ exposure and BPD^e^		
No O_2_ exposure, no BPD	1.00	reference
<200 hr O_2_, no BPD	0.17	1.19 (1.10–1.29)
≥200 hr O_2_, no BPD	0.58	1.79 (1.46–2.20)
BPD^f^	0.28	1.33 (0.80–2.19)
Maternal age, y		
<18	0.09	1.10 (0.96–1.25)
18–34	1.00	reference
≥35	−0.11	0.90 (0.86–0.93)

BPD = bronchopulmonary dysplasia.

^a^Classification of race/ethnicity in this study was based on the race of infants' mothers, as self-reported to the birth certificate clerk interviewing them at the Kaiser Permanente hospitals described in this study. Race was assessed in this study because of the well-known differences in RSV severity by race.

^b^Based on the algorithm of Brenner WE, et al. [19].

^c^See text for list of included International Classification of Diseases codes.

^d^Ascertained by electronic scanning of parental records, which included encounters and diagnoses identified in the Significant Problem List by the parent’s physician.

^e^Oxygen exposure, in hours, during the birth hospitalization. See text for details.

^f^All infants with BPD had ≥200 hours of oxygen exposure during the neonatal period.

The relative contribution of predictors to the overall predictive ability of our model was as follows: gender accounted for 36.0%, while presence of a sibling <5 years of age accounted for 28.5%, race/ethnicity accounted for 11.3%, and GA accounted for 10.1%. The contribution of other predictors was <7% each (see Additional file 1: Table S5). In multivariable analyses, predictors for having any episode were similar to predictors for having an episode lasting > 1 day (Table 4; see also Additional file 1: Table S5). Results for both multivariate models were similar for the sensitivity analysis cohort (see Additional file 1: Table S2 and Additional file 1: Table S3).

**Table 4:** Effect of demographic and clinical predictors on eventual bronchiolitis episodes >1 day in multivariate analysis

**Clinical and demographic predictors**	**Beta**	**Odds Ratio (95% CI)**
Sex (Male)	0.38	1.47 (1.40–1.54)
Race^a^		
White	1.00	reference
African-American	0.01	1.01 (0.93–1.10)
Asian	−0.17	0.85 (0.79–0.90)
Hispanic	0.13	1.13 (1.07–1.20)
Other/unknown	−0.11	0.89 (0.82–0.98)
Gestational age, wk		
32–33	0.58	1.79 (1.53–2.09)
34–36	0.43	1.53 (1.41–1.66)
37	0.18	1.20 (1.10–1.31)
38–40	1.00	reference
≥41	−0.11	0.90 (0.84–0.96)
Small for gestational age^b^	0.32	1.37 (1.16–1.61)
Congenital anomaly present^c^	0.15	1.17 (1.08–1.27)
Family history of asthma^d^		
None	1.00	reference
Father only	0.24	1.27 (1.08–1.50)
Mother only	0.45	1.56 (1.41–1.72)
≥1 sibling <5 y of age in home	0.37	1.45 (1.39–1.52)
O_2_ exposure and BPD^e^		
No O_2_ exposure, no BPD	1.00	reference
<200 hr O_2_, no BPD	0.22	1.25 (1.12–1.39)
≥200 hr O_2_, no BPD	0.88	2.41 (1.89–3.07)
BPD^f^	0.69	1.99 (1.13–3.49)
Maternal age, y		
<18	0.03	1.03 (0.85–1.26)
18–34	1.00	reference
≥35	−0.09	0.91 (0.86–0.96)

BPD = bronchopulmonary dysplasia.

^a^Classification of race/ethnicity in this study was based on the race of infants' mothers, as self-reported to the birth certificate clerk interviewing them at the Kaiser Permanente hospitals described in this study. Race was assessed in this study because of the well-known differences in RSV severity by race.

^b^Based on the algorithm of Brenner WE, et al. [19].

^c^See text for list of included International Classification of Diseases codes.

^d^Ascertained by electronic scanning of parental records, which included encounters and diagnoses identified in the Significant Problem List by the parent’s physician.

^e^Oxygen exposure, in hours, during the birth hospitalization. See text for details.

^f^All infants with BPD had ≥200 hours of oxygen exposure during the neonatal period.

## Discussion

Our study shows that bronchiolitis care episodes were frequent during the first two years of life in this study cohort and that, consistent with prior research, most occurred outside the hospital setting. In our large cohort, the duration of bronchiolitis episodes ranged from 1 to 27 days. Among episodes with duration >1 day, the mean duration was 7.0 ± 5.9 days, potentially indicating significant morbidity for this subset of infants. Preterm infants compared to full-term infants had higher overall bronchiolitis episode rates as well as higher rates of episodes with an ED and/or inpatient hospital admission during the first two years of life, which might reflect both an intrinsically higher risk of morbidity in this population and/or an increased level of parent and provider concern regarding their medical needs. In addition to prematurity, male gender, neonatal oxygen use, family history of asthma, and African-American or Hispanic race/ethnicity were associated with a higher frequency of at least one episode and/or a higher frequency of an episode >1 day.

Consistent with previous studies, the overall burden of disease in this population was relatively high, with 16.2% of children in this study having a documented episode of bronchiolitis before age two. An episode of care approach in characterizing the utilization patterns of infants and children with bronchiolitis may be useful for health services researchers, physicians, and parents in understanding a broader healthcare burden of disease. Analyzing episodes of care may be more informative than reporting the rates of discrete bronchiolitis-related medical encounters.

Our study has several important limitations. First, not all infants with bronchiolitis seek medical care. This study reports frequency and associated characteristics of bronchiolitis that led to a clinical encounter, but is not a prospective study of the entire burden of illness from bronchiolitis that includes patient reports of symptoms [21]. Nevertheless, the study design provided a good approximation of the total health care utilization associated with bronchiolitis. Second, we ascertained bronchiolitis diagnoses using ICD-9 codes. To be considered an episode, a subject needed to have ≥1 diagnostic code specific for bronchiolitis or viral pneumonia attributable to pathogens typically causing bronchiolitis (Category A). Because bronchiolitis is a clinical diagnosis, it is possible that some cases of bronchiolitis were instead coded as “fever,” “wheeze,” or “lower respiratory infection.”However, it is also possible that some pneumonia attributed to pathogens typically causing bronchiolitis was not in fact bronchiolitis, and that some episodes of asthma, viral pneumonia or upper respiratory infection were coded as “bronchiolitis.” Therefore, it is likely that the total error from miscoding of bronchiolitis is relatively small. Third, we estimated membership based on a combination of membership and use status in each cohort month. Because infants with a higher level of use might tend to have higher frequency of seeking medical care, it is possible that this might bias the results towards higher estimates of bronchiolitis episodes. Since the sensitivity analysis including only infants with 24 months of documented membership did not show much difference from the primary analysis, the effect of membership status is likely to be minimal. Fourth, there were no reliable data on smoking for this analysis. Because smoking in the home is a major predictor of bronchiolitis episodes and because maternal smoking is associated with preterm birth and with race/ethnicity, it is possible that smoking might confound the relationship between GA and incidence of bronchiolitis. However, in our previous work we have found that the overall rate of smoking among mothers delivering at KPMCP is 4%–8% [22]. Therefore, we do not believe this has had a large impact on our results. Fifth, because rates of episodes and episodes with hospitalization might be impacted by participation in an integrated healthcare delivery system, it is possible that our results may not be generalizable to other healthcare systems. However, because the KPMCP provides medical care to a large portion of infants born in Northern California, our data represent an important estimate of overall care episodes in this population. Sixth, our data on race/ethnicity include only the categories White, African-American, Asian, Hispanic and other, and we do not have access to more detailed data. Therefore, we cannot report how more specific subgroups of race/ethnicity might alter bronchiolitis risk.

Despite these limitations, our research design offers the unique opportunity to study an entire birth cohort using linked data with relatively little loss to follow-up and to report the health care utilization related to bronchiolitis in this prospective cohort. Thus, we are able to provide a comprehensive description of burden of disease in this population, including frequency and severity of illness. In addition, the large sample size of our cohort has allowed us to identify many predictors associated with the disease outcome, which include gestational age, race/ethnicity, maternal age, small for GA, presence of congenital anomaly, family history of asthma, at least one sibling <5 years of age in the home, and degree of oxygen exposure or having BPD.

## Conclusions

In this cohort, 16.2% of infants had one or more episodes of bronchiolitis in the first two years after birth, with episodes lasting from 1 to 27 days. Preterm infants were more likely to have a bronchiolitis episode compared to term infants and were more likely than term infants to have longer periods of healthcare utilization, perhaps reflecting both the medical needs of this particular high-risk population group and the vulnerability perceived by providers and parents. Using an episode of care approach to study the epidemiology of bronchiolitis may provide additional information about burden of disease for both preterm and term infants.

## Competing interests

Dr. Anthony Masaquel is an employee of MedImmune, LLC, the company which funded the study reported here.

## Authors’ contributions

VF participated in the conception and design of this study, participated in the analysis of the data, drafted the article, and has given final approval for the version to be published. AR participated in the conception and design of this study, participated in the analysis of the data, revised the manuscript critically for important intellectual content, and has given final approval for the version to be published. SXL participated in the conception and design of this study, analyzed the data, revised the manuscript critically for important intellectual content, and has given final approval for the version to be published. PK participated in data analysis, revised the manuscript critically for important intellectual content, and has given final approval for the version to be published. AM conceived and designed this study, revised the manuscript critically for important intellectual content, and has given final approval for the version to be published. GE conceived and designed this study, participated in the analysis of the data, revised the manuscript critically for important intellectual content, and has given final approval for the version to be published. All authors read and approved the final manuscript.

## Pre-publication history

The pre-publication history for this paper can be accessed here:

http://www.biomedcentral.com/1472-6963/12/144/prepub

## Supplementary Material

Additional file1 Online-Only Material Sensitivity Analyses Click here for file
