# Supplementary material for: Frequency, duration and predictors of bronchiolitis episodes of care among infants ≥32 weeks gestation in a large integrated healthcare system: a retrospective cohort study
Source: BMC Health Serv Res. 2012 Jun 8;12:144. doi: 10.1186/1472-6963-12-144 (PMC3407513; doi:10.1186/1472-6963-12-144)
Supplement: Additional file1 — Online-Only Material Sensitivity Analyses [file 1472-6963-12-144-S1.doc]

**Online-Only Material**

**Sensitivity Analyses**

**eTable 1. Descriptive comparison of cohorts with 20/24 and 24/24 months membership in first 2 y**ears

|  | **Cohort with 20/24 months membership** | | | **Cohort with 24/24 months membership** | | |
| --- | --- | --- | --- | --- | --- | --- |
|  | **Total, n (%)** | **Any episode, n (%)** | **Episode >1 day, n (%)** | **Total, n (%)** | **Any episode, n (%)** | **Episodes >1 day, n (%)** |
| Male sex | 63,054 (51.2) | 11,730 (58.7) | 5206 (60.1) | 61,076 (51.1) | 11,312 (58.6) | 5024 (60.0) |
| Race^a^ |  |  |  |  |  |  |
| White | 52,609 (42.7) | 8305 (41.6) | 3754 (43.4) | 51,119 (42.8) | 8029 (41.6) | 3634 (43.4) |
| African-American | 10,374 (8.4) | 1866 (9.3) | 740 (8.6) | 9959 (8.3) | 1775 (9.2) | 700 (8.4) |
| Asian | 24,977 (20.3) | 3599 (18.0) | 1505 (17.4) | 24,373 (20.4) | 3507 (18.2) | 1460 (17.5) |
| Hispanic | 25,619 (20.8) | 4704 (23.6) | 2047 (23.6) | 24,738 (20.7) | 4541 (23.5) | 1979 (23.7) |
| Other/unknown | 9685 (7.9) | 1494 (7.5) | 612 (7.1) | 9374 (7.8) | 1445 (7.5) | 595 (7.1) |
| Gestational age, wk |  |  |  |  |  |  |
| 32–33 | 1649 (1.3) | 406 (2.0) | 218 (2.5) | 1608 (1.3) | 397 (2.1) | 214 (2.6) |
| 34–36 | 7969 (6.5) | 1622 (8.1) | 830 (9.6) | 7747 (6.5) | 1581 (8.2) | 805 (9.6) |
| 37 | 7855 (6.4) | 1400 (7.0) | 632 (7.3) | 7621 (6.4) | 1342 (7.0) | 605 (7.2) |
| 38–40 | 86,708 (70.3) | 13,701 (68.6) | 5834 (67.4) | 84,093 (70.3) | 13,225 (68.5) | 5638 (67.4) |
| ≥41 | 19,083 (15.5) | 2839 (14.2) | 1144 (13.2) | 18,494 (15.5) | 2752 (14.3) | 1106 (13.2) |
| Small for gestational age^b^ | 1763 (1.4) | 323 (1.6) | 168 (1.9) | 1707 (1.4) | 311 (1.6) | 161 (1.9) |
| Congenital anomaly present^c^ | 8646 (7.0) | 1642 (8.2) | 764 (8.8) | 8387 (7.0) | 1571 (8.1) | 729 (8.7) |
| Family history of asthma^d^ |  |  |  |  |  |  |
| None | 116,763 (94.7) | 18,618 (93.2) | 8011 (92.5) | 113,264 (94.7) | 17,993 (93.2) | 7741 (92.5) |
| Father only | 1824 (1.5) | 336 (1.7) | 160 (1.9) | 1782 (1.5) | 325 (1.7) | 157 (1.9) |
| Mother only | 4561 (3.7) | 989 (5.0) | 476 (5.5) | 4405 (3.7) | 954 (4.9) | 459 (5.5) |
| Both parents | 116 (0.1) | 25 (0.1) | 11 (0.1) | 112 (0.1) | 25 (0.1) | 11 (0.1) |
| ≥1 sibling <5 y of age in home | 44,235 (35.9) | 8405 (42.1) | 3801 (43.9) | 43,053 (36.0) | 8158 (42.3) | 3693 (44.1) |
| Oxygen exposure and BPD^e^ |  |  |  |  |  |  |
| No oxygen exposure, no BPD | 118,599 (96.2) | 18,941 (94.9) | 8126 (93.9) | 115,032 (96.2) | 18,302 (94.8) | 7852 (93.8) |
| <200 hr O_2_, no BPD | 4117 (3.3) | 867 (4.3) | 427 (4.9) | 4000 (3.4) | 838 (4.3) | 413 (4.9) |
| ≥200 hr O_2_, no BPD | 466 (0.4) | 138 (0.7) | 89 (1.0) | 453 (0.4) | 135 (0.7) | 87 (1.0) |
| BPD^f^ | 82 (0.1) | 22 (0.1) | 16 (0.2) | 78 (0.1) | 22 (0.1) | 16 (0.2) |

BPD=bronchopulmonary dysplasia.

^a^Classification of race/ethnicity in this study was based on the race of infants' mothers, as self-reported to the birth certificate clerk interviewing them at the Kaiser Permanente hospitals described in this study. Race was assessed in this study because of the well-known differences in bronchiolitis severity by race.

^b^Based on the algorithm of Brenner WE, et al.[19]

^c^See text for list of included International Classification of Diseases codes.

^d^Ascertained by electronic scanning of parental records, which included encounters and diagnoses identified in the Significant Problem List by the parent’s physician.

^e^Oxygen exposure, in hours, during the birth hospitalization. See text for details.

^f^All infants with BPD had ≥200 hours of oxygen exposure during the neonatal period.

**eTable 2. Logistic regression results when outcome is any bronchiolitis episode**

|  | **Cohort with 20/24 months membership**  **(Number of outcomes=19,968)** | | | **Cohort with 24/24 months membership**  **(Number of outcomes=19,297)** | | |
| --- | --- | --- | --- | --- | --- | --- |
| **Clinical and demographic predictors** | **Beta** | **Odds Ratio (95% CI)** |  | **Beta** | **Odds Ratio (95% CI)** |  |
| Male sex | 0.36 | 1.44 (1.39–1.48) |  | 0.36 | 1.43 (1.39–1.48) |  |
| Race^a^ |  |  |  |  |  |  |
| White | Reference |  |  | Reference |  |  |
| African-American | 0.17 | 1.19 (1.12–1.25) |  | 0.17 | 1.18 (1.12–1.25) |  |
| Asian | −0.10 | 0.91 (0.87–0.95) |  | −0.09 | 0.91 (0.87–0.95) |  |
| Hispanic | 0.18 | 1.20 (1.15–1.25) |  | 0.19 | 1.21 (1.16–1.26) |  |
| Other/unknown | −0.01 | 0.99 (0.93–1.05) |  | 0.0 | 1.00 (0.94–1.06) |  |
| Gestational age, wk |  |  |  |  |  |  |
| 32–33 | 0.45 | 1.57 (1.39–1.77) |  | 0.46 | 1.59 (1.41–1.79) |  |
| 34–36 | 0.27 | 1.32 (1.24–1.40) |  | 0.28 | 1.33 (1.25–1.41) |  |
| 37 | 0.14 | 1.15 (1.08–1.22) |  | 0.13 | 1.14 (1.07–1.21) |  |
| 38–40 | Reference |  |  | Reference |  |  |
| 41+ | −0.06 | 0.95 (0.90–0.99) |  | −0.05 | 0.95 (0.91–0.99) |  |
| Small for gestational age^b^ | 0.16 | 1.17 (1.03–1.32) |  | 0.15 | 1.16 (1.03–1.32) |  |
| Congenital anomaly present^c^ | 0.13 | 1.14 (1.08–1.21) |  | 0.12 | 1.12 (1.06–1.19) |  |
| Family history of asthma^d^ |  |  |  |  |  |  |
| None | Reference |  |  | Reference |  |  |
| Father only | 0.16 | 1.17 (1.04–1.32) |  | 0.15 | 1.16 (1.03–1.31) |  |
| Mother only | 0.37 | 1.45 (1.35–1.56) |  | 0.37 | 1.45 (1.35–1.56) |  |
| Both parents | 0.38 | 1.46 (0.94–2.29) |  | 0.43 | 1.54 (0.98–2.41) |  |
| ≥1 sibling <5 y of age in home | 0.33 | 1.39 (1.35–1.44) |  | 0.33 | 1.39 (1.35–1.44) |  |
| Oxygen exposure and BPD^e^ |  |  |  |  |  |  |
| No oxygen exposure, no BPD | Reference |  |  | Reference |  |  |
| <200 hr O_2_, no BPD | 0.17 | 1.19 (1.10–1.29) |  | 0.17 | 1.19 (1.09–1.29) |  |
| ≥200 hr O_2_, no BPD | 0.58 | 1.79 (1.46–2.20) |  | 0.60 | 1.83 (1.486–2.25) |  |
| BPD^f^ | 0.28 | 1.33 (0.80–2.19) |  | 0.36 | 1.44 (0.87–2.39) |  |
| Maternal age, y |  |  |  |  |  |  |
| <18 | 0.09 | 1.10 (0.96–1.25) |  | 0.07 | 1.08 (0.94–1.24) |  |
| 18–34 | Reference |  |  | Reference |  |  |
| ≥35 | −0.11 | 0.90 (0.86–0.93) |  | −0.10 | 0.90 (0.87–0.93) |  |

BPD=bronchopulmonary dysplasia.

^a^Classification of race/ethnicity in this study was based on the race of infants' mothers, as self-reported to the birth certificate clerk interviewing them at the Kaiser Permanente hospitals described in this study. Race was assessed in this study because of the well-known differences in bronchiolitis severity by race.

^b^Based on the algorithm of Brenner WE, et al.[19]

^c^See text for list of included International Classification of Diseases codes.

^d^Ascertained by electronic scanning of parental records, which included encounters and diagnoses identified in the Significant Problem List by the parent’s physician.

^e^Oxygen exposure, in hours, during the birth hospitalization. See text for details.

^f^All infants with BPD had ≥200 hours of oxygen exposure during the neonatal period.

**eTable 3. Logistic regression results when outcome is bronchiolitis episodes >1 day duration**

|  | **Cohort with 20/24 months membership**  **(Number of outcomes=8658)** | | | **Cohort with 24/24 months membership**  **(Number of outcomes=8368)** | | |
| --- | --- | --- | --- | --- | --- | --- |
| **Clinical and demographic predictors** | **Beta** | **Odds Ratio (95% CI)** |  | **Beta** | **Odds Ratio (95% CI)** |  |
| Male sex | 0.38 | 1.47 (1.40–1.54) |  | 0.38 | 1.47 (1.40–1.53) |  |
| Race^a^ |  |  |  |  |  |  |
| White | Reference |  |  | Reference |  |  |
| African-American | 0.01 | 1.01 (0.93–1.10) |  | 0.0 | 1.00 (0.92–1.09) |  |
| Asian | −0.17 | 0.85 (0.79–0.90) |  | −0.17 | 0.84 (0.79–0.90) |  |
| Hispanic | 0.13 | 1.13 (1.07–1.20) |  | 0.13 | 1.14 (1.08–1.21) |  |
| Other/unknown | −0.11 | 0.89 (0.82–0.98) |  | −0.10 | 0.90 (0.83–0.99) |  |
| Gestational age, wk |  |  |  |  |  |  |
| 32–33 | 0.58 | 1.79 (1.53–2.09) |  | 0.60 | 1.81 (1.55–2.12) |  |
| 34–36 | 0.43 | 1.53 (1.41–1.66) |  | 0.43 | 1.53 (1.41–1.66) |  |
| 37 | 0.18 | 1.20 (1.10–1.31) |  | 0.17 | 1.19 (1.09–1.30) |  |
| 38–40 | Reference |  |  | Reference |  |  |
| 41+ | −0.11 | 0.90 (0.84–0.96) |  | −0.11 | 0.90 (0.84–0.96) |  |
| Small for gestational age^b^ | 0.32 | 1.37 (1.16–1.61) |  | 0.31 | 1.36 (1.15–1.61) |  |
| Congenital anomaly present^c^ | 0.15 | 1.17 (1.08–1.27) |  | 0.14 | 1.15 (1.05–1.25) |  |
| Family history of asthma^d^ |  |  |  |  |  |  |
| None | Reference |  |  | Reference |  |  |
| Father only | 0.24 | 1.27 (1.08–1.50) |  | 0.25 | 1.28 (1.09–1.51) |  |
| Mother only | 0.45 | 1.56 (1.41–1.72) |  | 0.45 | 1.56 (1.41–1.73) |  |
| Both parents | 0.35 | 1.42 (0.76–2.66) |  | 0.39 | 1.48 (0.79–2.77) |  |
| ≥1 sibling <5 y of age in home | 0.37 | 1.45 (1.39–1.52) |  | 0.38 | 1.46 (1.39–1.52) |  |
| Oxygen exposure and BPD^e^ |  |  |  |  |  |  |
| No oxygen exposure, no BPD | Reference |  |  | Reference |  |  |
| <200 hr O_2_, no BPD | 0.22 | 1.25 (1.12–1.39) |  | 0.22 | 1.24 (1.11–1.39) |  |
| ≥200 hr O_2_, no BPD | 0.88 | 2.41 (1.89–3.07) |  | 0.90 | 2.45 (1.92–3.14) |  |
| BPD^f^ | 0.69 | 1.99 (1.13–3.49) |  | 0.76 | 2.15 (1.22–3.79) |  |
| Maternal age, y |  |  |  |  |  |  |
| <18 | 0.03 | 1.03 (0.85–1.26) |  | −0.01 | 0.99 (0.80–1.22) |  |
| 18–34 | Reference |  |  | Reference |  |  |
| ≥35 | −0.09 | 0.91 (0.86–0.96) |  | −0.09 | 0.92 (0.87–0.97) |  |

BPD=bronchopulmonary dysplasia.

^a^Classification of race/ethnicity in this study was based on the race of infants' mothers, as self-reported to the birth certificate clerk interviewing them at the Kaiser Permanente hospitals described in this study. Race was assessed in this study because of the well-known differences in bronchiolitis severity by race.

^b^Based on the algorithm of Brenner WE, et al.[19]

^c^See text for list of included International Classification of Diseases codes.

^d^Ascertained by electronic scanning of parental records, which included encounters and diagnoses identified in the Significant Problem List by the parent’s physician.

^e^Oxygen exposure, in hours, during the birth hospitalization. See text for details.

^f^All infants with BPD had ≥200 hours of oxygen exposure during the neonatal period.

| **Episode Length, d** | **Gestational Age at Birth, wk** | **Birth Weight, g** | **Significant Comorbid Conditions** |
| --- | --- | --- | --- |
| 61 | 39 | 3335 | Yes |
| 62 | 37 | 3030 | No |
| 64 | 36 | 1570 | Yes |
| 67 | 38 | 3530 | No |
| 76 | 40 | 3847 | No |
| 76 | 38 | 3300 | Yes |
| 78 | 38 | 3295 | Yes |
| 89 | 34 | 2380 | Yes |
| 97 | 39 | 3350 | No |
| 134 | 35 | 2170 | Yes |
| 968 | 40 | 4700 | Yes |

**eTable 4. Description of infants with episodes >60 days duration excluded from the cohort**

**eTable 5. Relative contribution of clinical and demographic predictors**

| **Predictor** | **Any episode, %** | **Episodes >1 day, %** |
| --- | --- | --- |
| Sex | 35.99 | 29.48 |
| Race^a^ | 11.27 | 7.78 |
| Gestational age | 10.13 | 17.91 |
| Small for gestational age^b^ | 0.41 | 1.37 |
| Congenital anomaly present^c^ | 1.32 | 1.37 |
| Family history of asthma^d^ | 6.77 | 8.03 |
| ≥1 sibling <5 y of age in home | 28.50 | 26.90 |
| Oxygen exposure and BPD^e^ | 3.05 | 5.89 |
| Maternal age | 2.56 | 1.28 |

BPD=bronchopulmonary dysplasia.

^a^Classification of race/ethnicity in this study was based on the race of infants' mothers, as self-reported to the birth certificate clerk interviewing them at the Kaiser Permanente hospitals described in this study. Race was assessed in this study because of the well-known differences in bronchiolitis severity by race.

^b^Based on the algorithm of Brenner WE, et al.[19]

^c^See text for list of included International Classification of Diseases codes.

^d^Ascertained by electronic scanning of parental records, which included encounters and diagnoses identified in the Significant Problem List by the parent’s physician.

^e^Oxygen exposure, in hours, during the birth hospitalization. See text for details.
